# Genome-Wide DNA Methylation and Restriction-Modification Systems in *Casimicrobium huifangae* SJ-1, One of the Core Bacteria in Activated Sludge

**DOI:** 10.3390/microorganisms14081666

**Published:** 2026-07-30

**Authors:** Jihong Yi, Kaiyue Zhu, Ze-Shen Liu, Meng Si, Hong Sun, Haiyan Huang, He Jiang, Shuang-Jiang Liu, Shuning Wang

**Affiliations:** 1 State Key Laboratory of Microbial Technology, Microbial Technology Institute, Shandong University, Qingdao 266237, China; jihongyi@sdu.edu.cn (J.Y.); 18252759628@163.com (K.Z.); 15100165972@163.com (M.S.); shsdauwine1@163.com (H.S.); jianghe@sdu.edu.cn (H.J.); liusj@sdu.edu.cn (S.-J.L.); 2 State Key Laboratory of Microbial Diversity and Innovative Utilization, Environmental Microbiology Research Center, Institute of Microbiology, Chinese Academy of Sciences, Beijing 100101, China; liuzs@im.ac.cn; 3 Department of Pathogen Biology, School of Clinical and Basic Medicine, Shandong First Medical University & Shandong Academy of Medical Sciences, Jinan 250117, China; yanhh2006@163.com

**Keywords:** type I restriction-modification system, DNA methyltransferase, targeting motifs, host defense system, genetic transformation

## Abstract

*Casimicrobium huifangae* SJ-1 is one of the 28 core microbial groups in the activated sludge of municipal wastewater treatment plants. It exhibits strong environmental adaptability and interactions with other microbes. To develop a genetic manipulation system for this strain, we tested several broad-host-range plasmids, and pBBR1MCS-2 was successfully transformed into strain SJ-1. However, only the plasmid extracted from the transformant could be retransformed into strain SJ-1, suggesting strong host defense systems. We investigated the genome-wide DNA methylation and obtained a DNA methylation map and nine methylation-targeting motifs from *C. huifangae*. A modified pBBR1MCS-2 lacking all targeting motifs achieved transformation efficiency comparable to that of the host-derived plasmid, implicating the restriction modification (R-M) barrier of *C. huifangae*. REBASE annotation revealed nine R-M systems in the genome, including two of Type I, six of Type II, and one of Type III. Quantitative PCR showed that the restriction endonuclease component of the Type I RM-C system was transcriptionally upregulated in the transformant harboring pBBR1MCS-2. The subunits responsible for the methyltransferase and endonuclease functions of RM-C were expressed in *Escherichia coli*, purified, and characterized. The targeting motif of RM-C was implicated as 5′-GAGNNNNNNNTGCT-3′ based on in vitro cleavage assays and SMRT methylation calls. These findings reveal the DNA methylation characteristics of *C. huifangae* SJ-1 and outline the possible methylation-based defense architecture, providing a reference for future genetic manipulation.

## 1. Introduction

DNA methylation is widespread in the genomes of both prokaryotes and eukaryotes and plays diverse roles in cellular processes [1,2]. Three primary types of methylation—N^6^-methyladenine (6mA), 5-methylcytosine (5mC), and N^4^-methylcytosine (4mC)—have been identified in prokaryotes, and are mediated by DNA methyltransferases [3,4]. These enzymes are often integral components of restriction-modification (R-M) systems, which represent the most prevalent defense mechanism in bacteria against foreign DNA [5,6]. Typical R-M systems comprise two enzymes, i.e., methyltransferases (MTases) and restriction endonucleases (REases). MTases protect the host genome by methylating adenine or cytosine bases within specific targeting sequences, whereas REases recognize the methylation status of DNA at targeting sequences and cleave unmethylated or improperly methylated DNA. Thus, bacteria can effectively distinguish between “self” and “non-self” DNA: they cleave invading foreign DNA while protecting their own genome by adding specific methylation modifications that prevent cleavage by the cognate REases [7]. Manipulation of R-M systems has proven effective in enhancing genetic transformation efficiency in bacteria [8,9]. R-M systems are classified into four types (I−IV) based on their subunit structure, cofactor requirements, and mechanism of action [6]. Beyond defense, certain solitary methyltransferases such as Dam and CcrM participate in critical cellular processes including DNA repair, transcriptional control, and cell cycle regulation [4,10]. Despite these advances, prokaryotic DNA methylation remains incompletely understood.

Third-generation sequencing technologies, represented by Single Molecule Real-Time (SMRT) sequencing [11] and Oxford Nanopore Technology (ONT) [12], provide powerful analytical tools for studying the defense mechanisms of R-M systems. They enable the genome-wide detection of DNA methylation and genome-wide, site-specific detection of modifications at the single-nucleotide level. The SMRT platform, with its polymerase kinetic detection integrated with machine learning-based modification calling, not only allows the high-throughput identification of complex methylation patterns, but also facilitates the prediction of novel methylation motifs. It has been widely used to sequence prokaryotic methylomes and map genome-wide epigenetic modifications. The REBASE database (http://rebase.neb.com) has recorded methylome data for over 4000 bacterial species [13], highlighting the diversity of R-M systems. In addition, the biochemical characterization of DNA methyltransferases of R-M systems has also advanced rapidly, with recent examples including the characterization of BlihIA from *Bacillus licheniformis*, which features redundancy in its *hsdS* gene copies [14]. However, a large number of methylation motifs remain to be paired with their cognate methyltransferases.

*Casimicrobium huifangae* SJ-1 was isolated from activated sludge in the Qinghe Wastewater Treatment Plant in Beijing, China [15]. It is the type strain of the genus *Casimicrobium* within the family Casimicrobiaceae of the Betaproteobacteria. It has been recognized as one of the “most-wanted” 28 core functional microbial groups in the activated sludge of municipal wastewater treatment plants (WWTPs). Strain SJ-1 exhibits strong environmental adaptability, stress resistance, and the ability to interact with other microbes. It efficiently removes carbon, nitrogen, and phosphorus from wastewater and displays notable resistance to various heavy metals and antibiotics. It is an obligately aerobic, Gram-negative, rod-shaped bacterium that lacks flagella or pili, and its genome (4.4 Mb) contains no plasmids. Genomic analysis predicts diverse metabolic capabilities, including complete pathways for degrading aromatic compounds such as benzoic acid and phenylacetic acid, as well as the ability to mineralize various carbon sources, including carbohydrates, aromatics, and short-chain fatty acids. It is therefore considered a promising chassis for environmental engineering in the treatment of complex wastewater. However, a genetic manipulation system for this strain has not been established, and its engineering potential for industrial applications remains to be explored.

In this study, we attempted to introduce broad-host-range plasmids into *C. huifangae* SJ-1. Most attempts failed except with pBBR1MCS-2. However, only the plasmid DNA extracted from the transformant could be retransformed into strain SJ-1, suggesting the presence of strong host defense systems. Using SMRT and ONT sequencing, we investigated the genome-wide methylation patterns of strain SJ-1 and predicted its R-M systems. Through qRT-PCR, heterologous expression of the MTase and REase, as well as in vitro restriction and methylation assays, we characterized a type I R-M system and its targeting motif. This study outlines the landscape of complex R-M systems and provides a basis for future genetic manipulation of this recalcitrant yet ecologically important bacterium.

## 2. Materials and Methods

### 2.1. Strains and Cultivation

*C. huifangae* SJ-1 was isolated from activated sludge in the Qinghe Wastewater Treatment Plant in Beijing, China and previously characterized [15]; *E. coli* GB05-dir was used for RecET recombination [16]; *E. coli* DH5α, BL21(DE3), and JM110 were obtained from Novagen (Darmstadt, Germany). All other strains used in this study are listed in Table S1. *C. huifangae* SJ-1 was cultured aerobically at 30 °C with shaking at 200 rpm. The growth medium used was 3 × R2A containing (per liter) 9.6 g of Reasoner’s 2A (R2A) powder and 0.5 g of proteose peptone. *Escherichia coli* was grown at 37 °C with shaking at 200 rpm in Luria–Bertani (LB) medium supplemented with kanamycin (50 μg/mL) when harboring the plasmid for MTase expression.

### 2.2. Plasmid Transformation

Six broad-host-range plasmids (Table S2) were tested for introduction into *C. huifangae* SJ-1 by chemical transformation and electroporation. For this purpose, the cells of *C. huifangae* SJ-1 were harvested when OD_600_ reached 0.4–0.6, and treated according to the respective transformation methods. The tested chemical transformation methods included the CaCl_2_ method [17], Inoue method [18], and the TSS method [19]. For electroporation transformation, the competent cells of strain SJ-1 were prepared by washing three times with cold wash buffers, placed on ice for 10 min, and used immediately. The four wash buffers tested were 15% glycerol solution, EB buffer (270 mM sucrose and 15% glycerol), PEB buffer (1 mM Na_2_HPO_4_, 270 mM sucrose, and 15% glycerol), and SMP buffer (1 mM Na_2_HPO_4_, 1 mM MgCl_2_, 270 mM sucrose and 15% glycerol). Electroporation was performed on ice using a Gene Pulser Xcell Electroporation system (Bio-Rad Laboratories, Hercules, CA, USA) with 0.1 cm gap cuvettes. The electroporation parameters tested ranged from 1.0 to 2.5 kV (in increments of 0.25 kV), 25 μF, and 100–600 Ω (in increments of 100 Ω). After transformation, the cells were recovered in 3 × R2A broth at 30 °C for 4–5 h and then spread onto 3 × R2A agar plates containing the corresponding antibiotics at the concentrations listed in Table S2 (kanamycin at 50 μg/mL for selection of SJ-1 transformants, the same concentration used for *E. coli* transformants harboring pBBR1MCS-2).

### 2.3. Whole-Genome Methylation Sequencing and Prediction of R-M Systems

*C. huifangae* SJ-1 was grown to exponential phase (OD_600_ = 0.6). Genomic DNA was extracted and purified using the QIAGEN Genomic DNA Kit (QIAGEN, Hilden, Germany). DNA purity and concentration were assessed using a NanoDrop One spectrophotometer (Thermo Fisher Scientific Inc., Waltham, MA, USA) and a Qubit 3.0 Fluorometer (Thermo Fisher Scientific Inc., Waltham, MA, USA), respectively, and integrity was verified by agarose gel electrophoresis.

For ONT sequencing, DNA fragments were size-selected using magnetic beads, subjected to damage repair and end-repair, and purified again. Barcodes were added using the EXP-NBD196 kit (Oxford Nanopore Technologies, Oxford, UK), and sequencing adapters were ligated using the SQK-LSK110 ligation kit (Oxford Nanopore Technologies, Oxford, UK). The library was quantified with a Qubit 3.0 Fluorometer and loaded onto an R 9.4.1 flow cell for sequencing on the PromethION platform. Methylated bases were identified from the raw Fast5 files using Tombo software (version 1.5.1) with the alternative model algorithm. The detection results classified sites as methylated, non-methylated, or uncertain. Methylation level of a site was calculated as the number of methylated reads divided by the total number of methylated and non-methylated reads.

For PacBio SMRT sequencing (Beijing Berry & Kang Biotechnology Co., Ltd., Beijing, China), HiFi reads were generated with a sequencing depth of 303× coverage. Basecalling was performed using the SMRT Link pipeline (smrtlink-release_25.4.0.278749; Pacific Biosciences, Menlo Park, CA, USA), and all HiFi reads were above Q20 with an accuracy > 99.9%. Methylation detection was performed using the ipdSummary tool (included in the SMRT Link package). Motif discovery and re-annotation were performed using MotifMaker (version 0.3.1) with default parameters.

Neighbor-joining phylogenetic analysis of the predicted methyltransferases in *C. huifangae* SJ-1 was performed using MEGA11 (version 11.0.13) [20].

### 2.4. Construction and Validation of the De-Immunized pBBR1MCS-2 Plasmid

To construct the de-immunized pBBR1MCS-2, the entire plasmid backbone was redesigned to eliminate all R-M targeting motifs of strain SJ-1. A total of 30 point mutations were introduced via synonymous substitutions in coding regions and single-nucleotide changes in non-coding regions. The complete mutant plasmid was commercially synthesized (Beijing Tsingke Biotechnology Co., Ltd., Beijing, China), propagated in *E. coli* JM110, and verified by Sanger sequencing. The full sequence is provided as Sequence S1 in the Supplementary Materials.

To examine whether the host R-M systems directly cleave plasmid DNA, crude cell lysates of *C. huifangae* SJ-1 were prepared. Cells were harvested at the early exponential phase (OD_600_ = 0.3–0.4), washed twice with lysis buffer (33 mM Tris-HCl, pH 8.0), and disrupted by high-pressure homogenization. The homogenate was centrifuged at 35,000× *g* for 30 min at 4 °C, and the supernatant was collected as crude cell lysate. The protein concentration of the lysate was determined by the Bradford method.

Degradation reactions were performed in a 20 μL mixture containing 33 mM Tris-HCl (pH 8.0), 2 mM DTT, 5 mM MgCl_2_, 100 ng/μL plasmid DNA (either wild-type pBBR1MCS-2 or de-immunized pBBR1MCS-2, both extracted from *E. coli*), and 0.5 μg/μL crude cell lysate. The reactions were incubated at 30 °C for 1 h and then mixed with loading buffer for analysis by electrophoresis on 1% agarose gels in TBE buffer.

For quantitative comparison of transformation efficiency, electroporation was performed under the optimized parameters (1.25 kV, 200 Ω, 25 μF) using 0.1 cm gap cuvettes and 15% glycerol as the wash buffer. A total of 200 ng plasmid DNA (either pBBR1MCS-2 extracted from the SJ-1-mcs2 transformant or de-immunized pBBR1MCS-2 extracted from *E. coli* JM110) was added to 30 μL of competent cells prepared as described above. After electroporation, cells were recovered in 3 × R2A broth at 30 °C for 4–5 h and spread onto 3 × R2A agar plates containing kanamycin (50 μg/mL). Transformation efficiency was calculated as CFU per μg of DNA. Three independent biological replicates were performed for each plasmid.

### 2.5. qRT-PCR Detection of Genes Related to the R-M System in C. huifangae SJ-1

qRT-PCR was performed to examine transcriptional differences in predicted MTase and REase encoding genes between the wild-type strain SJ-1 and the transformant SJ-1-mcs2 carrying plasmid pBBR1MCS-2. Wild-type *C. huifangae* SJ-1 and the transformant SJ-1-mcs2 were inoculated at 2% (*v*/*v*) into 50 mL of 3 × R2A liquid medium, with three biological replicates per group. Cells were harvested when the OD_600_ reached 0.6, and their RNA was extracted using the Bacterial RNA Extraction Kit (Vazyme, Nanjing, China). gDNA removal and cDNA synthesis were carried out using 5× All-In-One MasterMix with the AccuRT Genomic DNA Removal Kit (Applied Biological Materials, Richmond, BC, Canada). The resulting product was used as the template for qRT-PCR analysis. qRT-PCR was performed using TB Green Premix Ex Taq II (TaKaRa, Shiga, Japan) on a LightCycler 480 instrument (Roche, Basel, Switzerland). The primers used for qRT-PCR are listed in Table S3. The 16S rRNA gene was used as an internal reference. Three independent biological replicates were used for each strain, and each sample was measured in three technical replicates by qRT-PCR, after which the average threshold cycle (C_t_) was calculated for each sample. For statistical analysis, a two-tailed Student’s *t*-test was performed for each gene to compare transcript levels between the wild-type and the transformant [21]. To correct for multiple comparisons, the Benjamini–Hochberg false discovery rate (FDR) correction was applied to all *p*-values.

### 2.6. Heterologous Expression and Purification of MS Subunits and R Subunit of Type I RM-C System

The gene fragment encoding M (M.ChuSJ1ORFCP) and S (S.ChuSJ1ORFCP) subunits was amplified with the gDNA of *C. huifangae* SJ-1 as the template and f-C-*hsd*M/S and r-C-*hsd*M/S as primers (Table S3). Plasmid pET28b(+)-C-*hsd*M/S was constructed using the RecET recombination method [16] and introduced into *E. coli* BL21(DE3) by electroporation. PCR amplification was carried out using ApexHF HS DNA Polymerase FS (Accurate Biology, Changsha, China). Plasmid extraction and DNA purification were performed using kits from Tiangen Biotech (Beijing, China) and Vazyme Biotech (Nanjing, China), respectively. Restriction enzymes and ligases used for plasmid construction were obtained from Thermo Fisher Scientific (Shanghai, China).

To express the R subunit (ChuSJ1ORFCP), the coding sequence was amplified from SJ-1 genomic DNA using primers 11015-F and 11015-R (Table S3). The pTrc99a vector backbone was linearized by PCR with primers ptrc99a-F and ptrc99a-R (Table S3). Both fragments were then assembled using a homologous recombination kit (Vazyme Biotech, Nanjing, China) following the manufacturer’s instructions. The resulting plasmid pTrc99a-C-hsdR was introduced into *E. coli* BL21(DE3) by electroporation for protein expression.

The transformants harboring pET28b(+)-C-hsdM/S (kanamycin resistance) or pTrc99a-C-hsdR (ampicillin resistance) were grown in LB medium supplemented with the appropriate antibiotic (50 μg/mL kanamycin or 50 μg/mL ampicillin) at 37 °C and 180 rpm rotation. When the culture reached an OD_600_ of 0.6–0.8, protein expression was induced with 0.6 mM isopropyl β-D-1-thiogalactopyranoside (IPTG) overnight at 16 °C. Cells were harvested by centrifugation at 4000× *g* for 15 min at 4 °C and resuspended in lysis buffer (20 mM sodium phosphate buffer, 500 mM NaCl, 1 mM PMSF, pH 7.4). Cell disruption was performed by high-pressure homogenization (JNBIO, Guangzhou, China). The homogenate was immediately cooled on ice and centrifuged at 35,000× *g* for 1 h at 4 °C to remove cell debris. The supernatant was loaded onto a pre-equilibrated 5-mL HisTrap FF column (Cytiva, Shanghai, China) at a flow rate of 2.5 mL/min. The column was washed with 10–20 column volumes of wash buffer (20 mM sodium phosphate buffer, 500 mM NaCl, pH 7.4), followed by stepwise elution with increasing concentrations of imidazole in the same buffer. The MS complex was eluted at 100 mM imidazole, whereas the R subunit was eluted at 50–100 mM imidazole. The fractions containing targeting protein were pooled and concentrated by ultrafiltration with an Amicon centrifugal filter (50-kDa cutoff; Millipore, Burlington, MA, USA). The size and purity of the purified protein was determined by SDS-PAGE (10% gel; Epizyme, Shanghai, China) analysis, and the gels were stained with Coomassie brilliant blue [22].

### 2.7. In Vitro Restriction and Methylation Assays of the RM-C System

To generate reporter plasmids carrying a single copy of each candidate motif, the pET-28b(+) backbone was linearized by PCR using primers 28b-F and 28b-R. DNA fragments containing the desired motif sequences (motif 6-1, motif 6-2, motif 6-3, motif 6-4, or motif 8-1) were amplified from synthetic oligonucleotides using corresponding primer pairs (see Table S3 for all primer sequences). Each linearized backbone and its matching insert were assembled using a homologous recombination kit (Vazyme Biotech, Nanjing, China) following the manufacturer’s instructions. The resulting plasmids were designated as pMotif6-1, pMotif6-2, pMotif6-3, pMotif6-4, and pMotif8-1, respectively. All constructs were verified by Sanger sequencing (Supplementary Sequencing Data). The verified plasmids were then transformed into *E. coli* JM110 for propagation, and plasmid DNA was extracted and stored for subsequent assays.

All reactions were performed in a buffer containing 33 mM Tris (pH 8.0) and 2 mM DTT. The standard reaction mixture (15 μL) contained 200 ng of reporter plasmid, and where indicated, ATP (2 mM), MgCl_2_ (5 mM), and *S*-adenosyl-methionine (SAM, 1 mM). For all MSR experiments, the MS complex and R subunit were pre-incubated at a molar ratio of M_2_S:R = 1:2 at room temperature for 30 min to allow for assembly of the presumed active MSR complex. Because the enzyme concentrations were based on presumed stoichiometries, the actual molarity of active protein may be lower than the amounts added [23]. The concentrations of MS complex and R subunit are given as estimated molarities based on the presumed assembly states (M_2_S for MS and monomer for R). For restriction activity assays (subunit requirement, cofactor dependence, and motif validation), the pre-assembled MSR was added to the reaction at a final concentration of 20 nM (based on the presumed M_2_S:R stoichiometry). For subunit requirement experiments (MS alone, R alone), each component was added individually at 20 nM. The reaction mixtures were incubated at 30 °C for 20 min, after which the reactions were terminated by heating at 95 °C for 2 min, followed by centrifugation at 13,000× *g* for 2 min.

For the methylation protection assay, the pre-incubation step contained MS complex at 500 nM (as M_2_S) with or without 1 mM SAM in a 15 μL reaction, and was carried out at 30 °C for 90 min. Subsequently, pre-assembled MSR (20 nM, based on the presumed M_2_S:R stoichiometry) and ATP (2 mM) were added to the same tube, and the reaction was continued for another 20 min at 30 °C before termination as described above. All terminated samples were centrifuged at 12,000× *g* for 2 min to remove precipitated protein, and the supernatants were resolved by electrophoresis on 1% agarose gels in TBE buffer.

## 3. Results and Discussion

### 3.1. Transformation of Broad-Host-Range Plasmids into C. huifangae SJ-1

Based on the characteristics of *C. huifangae* SJ-1 Gram-negative, non-flagellated, and lacking pili—we tested introducing six broad-host-range plasmids (Table S2) into the cells of strain SJ-1 by chemical transformation and electroporation. These plasmids harbor different replicons and have been shown to replicate in a wide variety of Gram-negative bacteria. However, most of them failed to be transformed into strain SJ-1 despite various optimization attempts in our study. Only when we tested pBBR1MCS-2 did we obtain a few transformants via electroporation, one of which, designated SJ-1-mcs2, was found to stably maintain the plasmid. This transformant was further verified by sequencing its 16S rRNA gene and amplifying a specific fragment of pBBR1MCS-2 using the universal primers M13F/M13R (Figure S1). However, in repeated experiments, we found that *C. huifangae* SJ-1 could only accept the pBBR1MCS-2 plasmids extracted from the transformant SJ-1-mcs2, whereas the plasmid DNA extracted from *E. coli* DH5α or JM110 was blocked, suggesting the presence of strong host defense systems in strain SJ-1. Using the plasmid DNA extracted from the transformant, we further determined the optimal electroporation parameters for transformation (1.25 kV, 200 Ω, and 25 μF with 15% glycerol solution as buffer), achieving an efficiency of approximately 1.2 × 10^5^ CFU/μg DNA.

### 3.2. Investigation of the Whole-Genome Methylation of C. huifangae SJ-1

Given that the R-M system represents the most prevalent defense mechanism in bacteria against foreign DNA, we used PacBio SMRT and ONT sequencing to obtain genome-wide methylation information for *C. huifangae* SJ-1. Statistical analysis of its methylation sites is shown in Table 1. *C. huifangae* SJ-1 exhibits distinctive methylation characteristics. Its average CpG dinucleotide methylation level is 14.7%, consistent with the general pattern of dynamic regulation in prokaryotic genomes. The high methylation level of Dam (70%) and Dcm (85%) suggests several possibilities. Dam and Dcm methyltransferases may play important roles during DNA synthesis, including regulating replication, repairing base mismatches and mutations, and influencing protein synthesis, indicating that the strain may possess complex epigenetic regulatory mechanisms. Additionally, dense methylation marks may serve as signals for the strain to recognize “self”. Although Dam/Dcm methyltransferases do not directly constitute an R-M system, they may cooperate with restriction-modification systems to form a defense barrier. This cooperation is exemplified by restriction endonucleases such as MboI and EcoRII, which cleave unmethylated foreign DNA at GATC and CCWGG sequences, respectively, while host DNA is protected by Dam and Dcm methylation, thereby enhancing environmental adaptability [24,25]. The widespread moderate levels of 6mA (24%) and 5mC (22%) modifications across the genome suggest the potential coexistence of multiple R-M systems or complex epigenetic regulation.

Since methylation levels vary among different modification types and across species, we analyzed the distribution of methylation levels for each type across the genome. As shown in Figure S2, the genome of strain SJ-1 was divided into 10 intervals (0–0.1, 0.1–0.2, 0.2–0.3, etc.), and the proportion of sites within each interval represents the methylation signature of the strain.

### 3.3. Prediction of the R-M Systems in C. huifangae SJ-1

**Figure 1:**
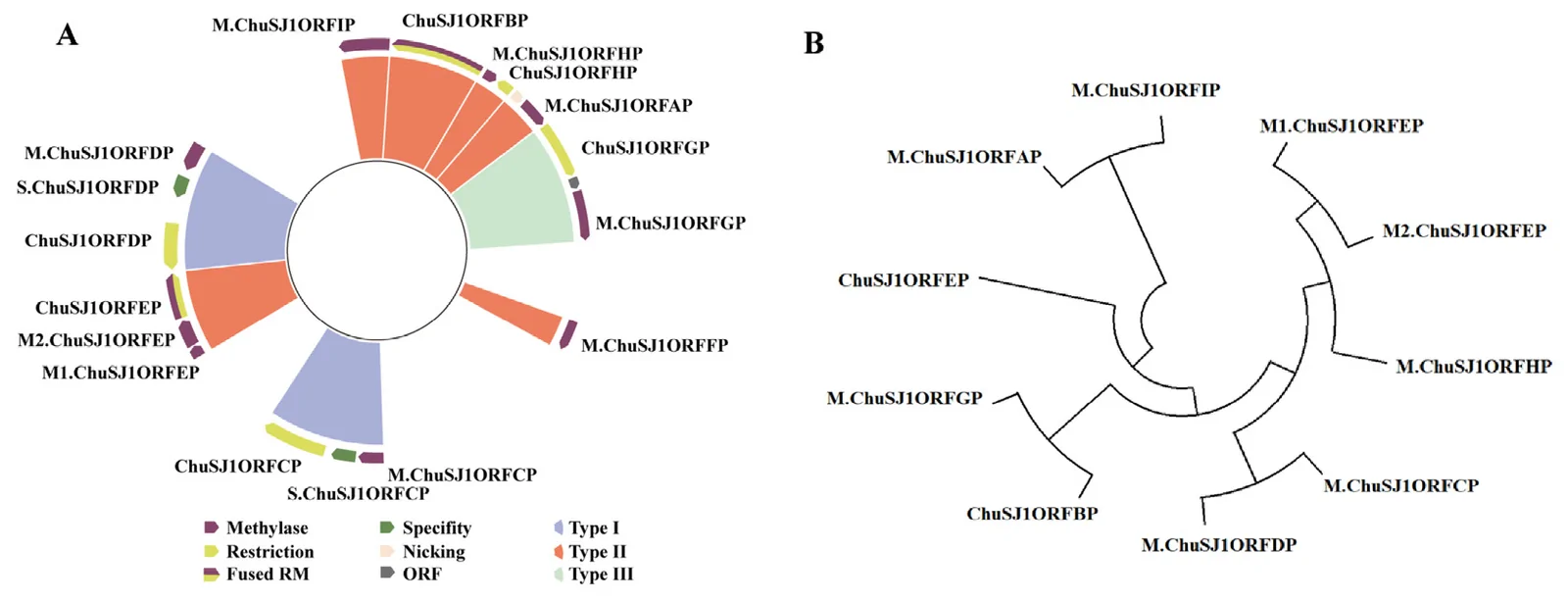
Genomic organization and phylogenetic analysis of the predicted R-M systems in *C. huifangae* SJ-1. (**A**) Genomic location and gene arrangement of the nine predicted R-M systems. (**B**) Neighbor-joining phylogenetic tree of the predicted methyltransferases from strain SJ-1.

REBASE is currently the most comprehensive open database, containing extensive information on REases, MTases, and related proteins. REBASE has already annotated the genome of *C. huifangae* SJ-1 and predicted nine potential R-M systems (Table 2 and Figure 1A), including two of Type I, six of Type II, and one of Type III. According to REBASE naming conventions, a putative REase or MTase typically has an uppercase “P” appended to their names (e.g., HindIII^P^) to indicate that their function has not been experimentally verified. Interestingly, there are two fused MTase-REase proteins (No. 3 and No. 4) and two orphan MTases (No. 7 and No. 8).

Typically, wild-type bacterial strains carry 2–5 R-M systems. The presence of nine R-M systems in *C. huifangae* SJ-1 may reflect the strong selective pressure exerted by foreign DNA in the activated sludge environment, where horizontal gene transfer and phage predation are frequent [26,27,28]. It is tempting to speculate that the diversity of R-M systems could contribute to the robust barrier against exogenous DNA observed in this strain, and may help explain why *C. huifangae* SJ-1 harbors no plasmids and contains only an incomplete prophage sequence. Using DefenseFinder (https://defensefinder.mdmlab.fr/), which contains 152 known anti-phage defense systems in prokaryotic genomes [29], we identified eight additional anti-phage defense systems in the genome of *C. huifangae* SJ-1 beyond the R-M systems predicted by REBASE (Table S4). It is reported that activated sludge systems support high densities of bacteriophages [30]. In response to this constant predation pressure, activated sludge bacteria have evolved diverse defense systems [31]. A recent long-term metagenomic study revealed that over 91% of bacterial genomes in activated sludge harbor at least one complete defense system, such as R-M and CRISPR-Cas, which exhibit a “pan-immunity” property [32]. Considering that strain SJ-1 serves as a “module hub” in the bacterial community of WWTPs, its multiple defense systems—including both R-M and other anti-phage systems—could potentially prevent invasion by harmful exogenous genes, especially from bacteriophages. Therefore, these defense systems could contribute to stabilizing the core bacterial community structure, ensuring the functional resilience of the wastewater treatment process.

We further performed evolutionary analysis of the annotated MTases, and a phylogenetic tree was constructed using the neighbor-joining method (Figure 1B). Both M.ChuSJ1ORFCP (MTase of RM-C, RM-C is named in Section 3.5) and M.ChuSJ1ORFDP are predicted members of the Type I R-M system and are located within the same branch. The MTase catalytic core domain of this system (e.g., the SAM-binding site) is highly conserved throughout evolution, resulting in high sequence similarity and clustering into a well-supported clade in the phylogenetic tree. In contrast, MTases of Type II R-M systems, which adapt to diverse substrate recognition requirements, exhibit significant sequence divergence in their DNA-binding domains. This leads to lower overall sequence similarity and distribution across multiple dispersed branches in the phylogenetic tree, with some subclasses even intermixing with Type I/III MTases. Traditional classification of MTases is mainly based on the enzyme’s molecular mechanism and composition rather than strictly on sequence similarity; therefore, evolutionary analysis can only assist in verifying the rationality of functional classification.

### 3.4. Analysis of the Methyltransferase-Specific Targeting Motifs in C. huifangae SJ-1

From the results of SMRT and ONT sequencing, we obtained nine MTase-specific targeting motifs in strain SJ-1 (Table 3). Based on motif characteristics, MTases can be preliminarily matched to their targeting motifs. Targeting motifs of different types of R-M systems have distinct features. For Type I R-M systems, the MTase recognizes bipartite asymmetric sequences composed of two specific half-sites separated by a non-specific spacer of defined length [33]. Accordingly, their targeting motifs typically appear as two sets of degenerate sequences that are reverse complements of each other, with the methylation site located within one of the half-sites. For conventional Type IIP systems, the MTase recognizes short, palindromic sequences (typically 4–8 bp) that are identical on both strands [34]. For Type IIG systems, the MTase and REase domains are fused within a single polypeptide chain, and these systems typically recognize asymmetric sequences [35]. For Type III R-M systems, the targeting sequences are also asymmetric and short (5–6 bp), but lack the bipartite structure characteristic of Type I [34]. Among the nine targeting motifs of strain SJ-1, two sets (four motifs: 5/6 and 7/8, each pair consists of reverse complements.) exhibited features of the Type I R-M system, and two motifs displayed complementary palindromic characteristics (motifs 2 and 9). Since we identified two Type I R-M systems (Table 2), they likely correspond to match the two sets of targeting motifs (5/6 and 7/8), respectively.

We further compared the specific targeting motifs found in *C. huifangae* SJ-1 with the sequence of plasmid pBBR1MCS-2. The results showed that six specific targeting motifs of the strain SJ-1 are present in plasmid pBBR1MCS-2 (Table S5), with the most frequent motif appearing 15 times. If a strain primarily relies on its R-M system to defend against foreign DNAs, the more specific targeting motifs present on an exogenous plasmid, the more likely it is to be recognized and cleaved by the host’s R-M system. This may explain why almost all the broad-host-range plasmids we tested failed to be introduced into strain SJ-1.

Given that conventional transformation attempts could not overcome this R-M barrier, we sought to systematically eliminate all R-M targeting motifs of strain SJ-1 from the pBBR1MCS-2 backbone. Guided by the motif profiles (Table S5), we rationally redesigned the plasmid using a three-pronged strategy: (i) for non-coding regions, single nucleotide substitutions were introduced to disrupt each targeting motif; (ii) for coding regions, synonymous codon replacements were used without altering the amino acid sequence; (iii) for the core replicon and essential functional elements, mutations were designed to avoid altering the secondary structure as predicted in silico, and their functionality was verified by confirming replication in *E. coli*. The complete sequence of the mutated plasmid is provided in the Supplementary Materials (Sequence S1). The resulting “de-immunized” mutant plasmid was synthesized and transformed into wild-type *C. huifangae* SJ-1. As shown in Figure S3A, using the optimized electroporation conditions described above, transformants were readily obtained and stably maintained under kanamycin selection.

We also performed an in vitro plasmid degradation assay using crude cell lysates of *C. huifangae* SJ-1. As shown in Figure S3B, the unmodified pBBR1MCS-2 plasmid extracted from *E. coli* was completely degraded upon incubation with the lysate, whereas the de-immunized plasmid remained intact under identical conditions. This result indicates that removal of the targeting motifs on the plasmid effectively protects it from restriction, most likely by host R-M systems. Furthermore, we compared the transformation efficiency of the de-immunized plasmid (extracted from *E. coli*) with that of the unmodified pBBR1MCS-2 plasmid extracted from the SJ-1-mcs2 transformant. Under identical experimental conditions, the de-immunized plasmid yielded a transformation efficiency (approximately 1.4 × 10^5^ CFU/μg DNA) comparable to that of the unmodified plasmid, with no statistically significant difference between the two (Figure S3C, Student’s *t*-test, ns). This result indicates that elimination of the R-M targeting motifs is sufficient to enable efficient plasmid transformation. These results demonstrate that the precise removal of all R-M targeting sites efficiently bypassed the host restriction barrier. A similar “stealth DNA” strategy has been reported [36]; our work validates that this approach can overcome the complex R-M systems of *C. huifangae* SJ-1.

### 3.5. Identification of a Type I R-M System in C. huifangae SJ-1 by qRT-PCR

**Figure 2:**
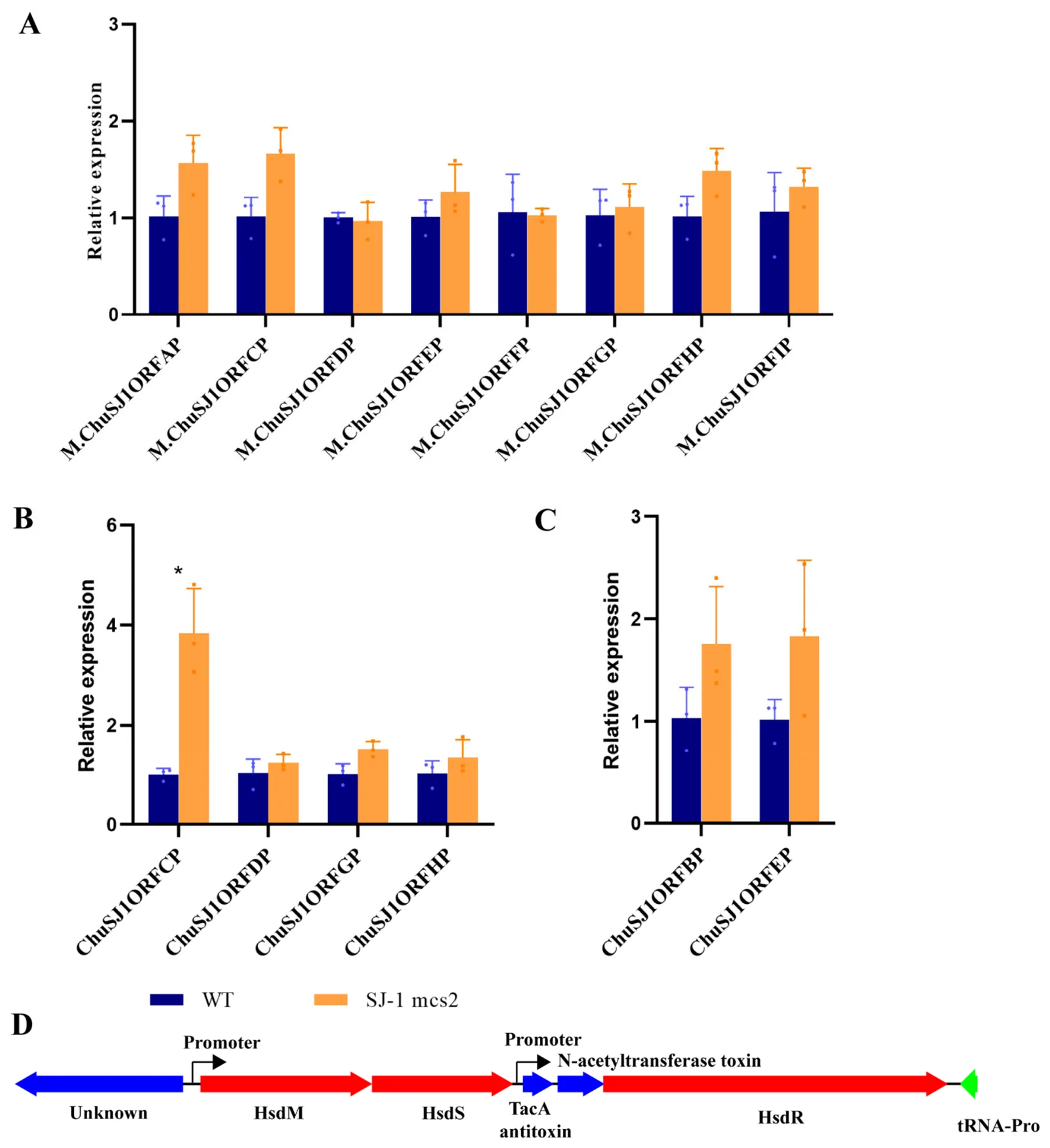
Transcriptional differences of R-M system-related genes between wild-type *C. huifangae* SJ-1 and the transformant *C. huifangae* SJ-1-mcs2, and the genomic organization of the RM-C system. (**A**) Transcriptional differences of MTase (MT) genes. (**B**) Transcriptional differences of REase (RE) genes. (**C**) Transcriptional differences of genes encoding bifunctional fusion proteins with both MTase and REase activities (MT/RE). (**D**) Genomic organization of the RM-C system. Arrows indicate the genes encoding the methyltransferase (HsdM), specificity subunit (HsdS), a pair of putative toxin–antitoxin (TA) modules, and the restriction endonuclease (HsdR). The HsdMS genes and the downstream region (TA + HsdR) are driven by separate promoters (depicted as bent arrows). Statistical significance: *, *p* < 0.05.

To examine whether the presence of pBBR1MCS-2 is associated with transcriptional changes in the predicted R-M system genes, we performed qRT-PCR to compare their transcript levels between the wild-type strain and the transformant SJ-1-mcs2. As shown in Figure 2, the mRNA levels of the REase gene (ChuSJ1ORFCP, FKL89_RS11015) of the Type I R-M system RM-C (encoded by FKL89_RS10995, FKL89_RS11000, and FKL89_RS11015; system No. 1 in Table 2) were significantly higher in the transformant, with a fold increase of 3.7 (adjusted *p* < 0.05, Benjamini–Hochberg correction). The MTase gene (M.ChuSJ1ORFCP, FKL89_RS10995) showed a fold increase of 1.6, but this difference did not reach statistical significance after correction for multiple comparisons. No significant changes were observed for genes belonging to other R-M systems.

Genomic analysis revealed an unusual feature of the RM-C gene cluster in strain SJ-1: a pair of putative toxin–antitoxin (TA) modules is inserted between the genes encoding the specificity subunit (HsdS) and the endonuclease subunit (HsdR) (Figure 2D). While TA modules are occasionally found within R-M clusters as part of so-called “defense islands”, the organization in strain SJ-1 displays several distinct characteristics. Unlike many defense islands that are transcribed under a common regulator (e.g., RptR) [37], the RM-C cluster in strain SJ-1 lacks an upstream *rptR* homolog. Moreover, the MS genes (*hsdM* and *hsdS*) and the downstream region containing the TA modules and *hsdR* appear to be driven by separate promoters, implying that the MS complex and the R subunit are transcribed independently—a rare architecture supported by our qRT-PCR data showing distinct upregulation folds for the MTase (1.6-fold) and REase (3.7-fold) genes (Figure 2A,B). The TA modules encode a predicted *N*-acetyltransferase toxin. Whether this unique gene organization contributes to the transcriptional upregulation of RM-C in the plasmid-bearing transformant is unknown and requires further investigation. Nevertheless, the observed upregulation of RM-C, together with its unique genomic structure, prompted us to investigate the biochemical activity of this system outside its native genomic context.

### 3.6. Characterization of the Type I RM-C System and Its Specific Targeting Motif

**Figure 3:**
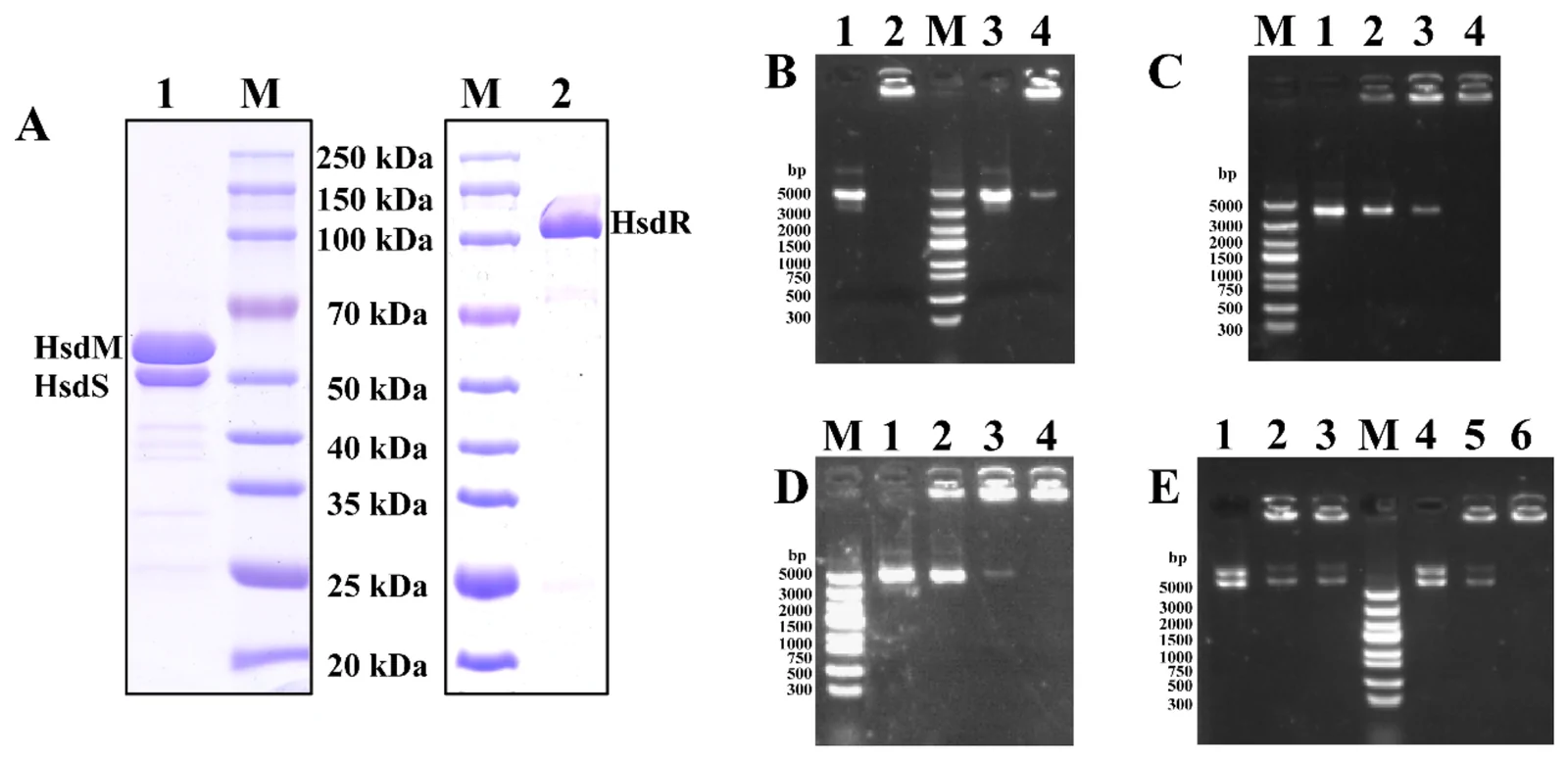
In vitro heterologous expression and functional characterization of the Type I RM-C system from *C. huifangae* SJ-1. (**A**) SDS-PAGE analysis of heterologously expressed subunits of the RM-C system. Lane M, protein marker; lane 1, purified MS complex (co-expressed HsdM and HsdS subunits); lane 2, purified R subunit (HsdR). (**B**) Identification of the cognate motif. pMotif6-1 (lanes 1–2) and pMotif8-1 (lanes 3–4) were incubated without or with the pre-assembled MSR complex. Only pMotif6-1 was completely degraded by MSR (lane 2). M, DNA marker. (**C**) Subunit requirement. pMotif6-1 was incubated with no protein (lane 1), MS alone (lane 2), R alone (lane 3), or MSR (lane 4). Cleavage occurred only when both MS and R were present (lane 4). (**D**) Cofactor dependence. Lane 1, negative control (no MSR); lane 2, reaction without ATP; lane 3, reaction without Mg^2+^; lane 4, complete reaction (with MSR, ATP and Mg^2+^). ATP is absolutely required, while Mg^2+^ greatly enhances activity. (**E**) Methylation protects the targeting site from cleavage. Lanes 1–3: pMotif6-1 pre-incubated with MS under different conditions, then sampled as methylation controls. Lane 1, pre-incubation without MS (control); lane 2, with MS + SAM; lane 3, with MS alone (no SAM). Lanes 4–6: after pre-incubation as in lanes 1–3 respectively, the reactions were further supplemented with pre-assembled MSR and ATP. Lane 4, no MSR added (control); Lane 5, MS + SAM pre-incubation + MSR + ATP; lane 6, MS alone pre-incubation + MSR + ATP. Only pre-incubation with MS plus SAM (lane 5) protected the plasmid from subsequent restriction.

To characterize the components of the Type I RM-C system, we heterologously expressed its three subunits. The M and S subunits were co-expressed in *E. coli* BL21(DE3) using pET28b(+)-C-hsdM/S, and the purified MS complex showed two bands on SDS-PAGE (Figure 3A) corresponding to HsdM (~60 kDa) and HsdS (~49 kDa). Densitometric analysis using ImageJ (version 2.18.0/1.54p; National Institutes of Health, Bethesda, MD, USA) revealed that the intensity ratio of the HsdM band to the HsdS band was approximately 1.8:1 after normalization by molecular weight, which is consistent with the presumed M_2_S stoichiometry. The R subunit (HsdR, ~118 kDa) was separately expressed using pTRC99a-C-hsdR and purified similarly. All three purified proteins were used for subsequent in vitro functional assays.

Using the purified MS complex and R subunit, we performed in vitro reconstitution assays to identify the possible targeting motif of the RM-C system. Unless otherwise stated, reactions containing the complete MSR complex were assembled with an M_2_S:R molar ratio of 1:2 and incubated at room temperature for 30 min.

According to the analysis in Section 3.4, motifs 5/6 and 7/8 in Table 3 exhibited the targeting motif features of the Type I R-M system. Based on motifs 6 and 8, we constructed two reporter plasmids, respectively, each carrying a single copy of one candidate motif: pMotif6-1 (5′-GAGATCCGGCTGCT-3′) and pMotif8-1 (5′-GAGGGGAATTG-3′). When incubated with the complete MSR complex, pMotif6-1 was completely degraded: no DNA band was visible in the gel, with only a faint residual signal remaining in the well, likely representing protein–DNA aggregates (Figure 3B, lane 2). In contrast, pMotif8-1 incubated under identical conditions yielded a visible DNA band, albeit weaker than the no-protein control (Figure 3B, lane 4), suggesting some non-specific binding. To assess whether the seven degenerate bases (N_7_) of motif 6 affect recognition, we constructed 2 additional variants (pMotif6-2 and pMotif6-3, Figure S4) with different N_7_ sequences. All three variants were equally degraded by the MSR complex (Figure S4, lanes 1–3), indicating that the RM-C system tolerates sequence variation in the spacer region. In contrast, a mutant in which the conserved 3′-terminal TGCT was replaced with AGCT (pMotif6-4) completely abolished cleavage (Figure S4, lane 4), demonstrating that the TGCT tetranucleotide is essential for recognition. These results implicate that motif 6 (Table 3) is the specific targeting sequence for the RM-C system.

To confirm that endonucleolytic activity requires the full MSR complex, pMotif6-1 was incubated with MS alone, R alone, or MSR. As shown in Figure 3C, neither MS nor R alone affected the supercoiled structure of the plasmid; only the combined MSR complex caused complete degradation. This is consistent with the well-characterized Type I system EcoKI from *E. coli*, in which HsdR, HsdM, and HsdS are all required for restriction activity, and the M_2_S_1_ complex alone possesses only a methyltransferase function [23]. Our results similarly demonstrate that both the methyltransferase (MS subunits) and the restriction endonuclease (R subunit) are necessary for cleavage.

We next examined cofactor requirements. Type I restriction enzymes typically depend on ATP and Mg^2+^. Omission of ATP completely abolished cleavage (Figure 3D, lane 2), while omission of Mg^2+^ reduced but did not eliminate activity, leaving a substantial fraction of plasmid intact (lane 3). The residual activity without added Mg^2+^ may be due to trace amounts of the metal ion bound to the enzyme during heterologous expression, as often observed during the purification of metal-dependent enzymes.

Finally, to verify that the MS complex functions as a methyltransferase that protects the targeting site, we performed a two-step protection assay. pMotif6-1 was pre-incubated with MS in the absence or presence of the methyl donor SAM for 90 min. Subsequently, the pre-assembled MSR complex and ATP were added, and the reaction was incubated for 20 min at 30 °C. As shown in Figure 3E, pre-incubation with MS plus SAM completely protected the plasmid from subsequent restriction, whereas pre-incubation with MS alone (without SAM) did not confer protection. A control without MSR showed no degradation. These results confirm that the MS complex methylates motif 6-1 in a SAM-dependent manner, and that this methylation blocks cleavage by the MSR complex.

Collectively, the in vitro reconstitution experiments and SMRT methylation calls implicate motif 6 (5′-GAGNNNNNNNTGCT-3′, Table 3) as the targeting sequence for the Type I RM-C system, with the methylated adenine inferred to be at position 2 (motif 6) and at the corresponding position on the complementary strand (motif 5, CenterPos = 4), where direct biochemical evidence for methylation is required through further study.

## 4. Conclusions

In this study, we employed PacBio SMRT and ONT sequencing to obtain the first complete genome-wide DNA methylation map of *C. huifangae* SJ-1, a core bacterial taxon in the activated sludge of WWTPs. From the sequencing data and REBASE, nine MTase-specific targeting motifs and nine R-M systems (two of Type I, six of Type II, and one of Type III) were predicted. The presence of multiple R-M systems may contribute to the strong barrier to exogenous DNA, as demonstrated by the failure of transforming most broad-host-range plasmids into this strain. By systematically eliminating all R-M targeting motifs of strain SJ-1 from the pBBR1MCS-2 backbone, we successfully constructed a “de-immunized” mutant plasmid that enabled efficient transformation. Furthermore, through qRT-PCR, heterologous expression, and in vitro biochemical assays, we characterized the Type I RM-C system and implicated its targeting motif (5′-GAGNNNNNNNTGCT-3′) based on in vitro cleavage assays and SMRT methylation calls.

However, several limitations of this study should be acknowledged. Among the nine predicted R-M systems, only RM-C was characterized; the other eight systems remain to be investigated. Furthermore, while nine R-M systems were predicted in the genome, their roles in foreign plasmid restriction remain unclear, as well as the eight predicted anti-phage defense systems. Future time-course experiments following plasmid introduction could provide a more direct assessment of the transcriptional dynamics of R-M systems and other defense systems in response to foreign DNA invasion. Nevertheless, our de-immunization experiments—including the protection of the de-immunized plasmid from degradation by crude cell lysates—implicate that R-M systems are a key factor in the transformation barrier. Further studies are needed to address these aspects. For the de-immunization strategy, we may extend it to construct genetic tools, such as CRISPR-based systems, to inactivate key R-M components (e.g., RM-C), thereby facilitating routine genetic manipulation of this recalcitrant strain. Despite these limitations, our findings provide new insights into the defense systems of a core player in wastewater treatment and lay the foundation for developing genetic manipulation strategies in this ecologically important bacterium.

## Figures and Tables

**Table 1:** Genome-wide methylation site statistics in *C. huifangae* SJ-1.

Type of Methylation	Number of Detected Sites ^e^	Average Methylation Rate ^f^
5mC (+) ^a^	1,389,792	22.301%
5mC (−)	1,391,109	
CpG (+) ^b^	521,242	14.747%
CpG (−)	521,155	
6mA (+)	835,649	24.399%
6mA (−)	837,852	
dcm (+) ^c^	8945	85.023%
dcm (−)	8945	
dam (+) ^d^	27,273	70.192%
dam (−)	27,272	

^a^ (+) indicates the positive strand, (−) indicates the negative strand. ^b^ CpG denotes the methylation at the C5 position of cytosine followed by a guanine, connected by a phosphate group. ^c^ dcm denotes sites modified by the site-specific DNA methyltransferase Dcm, which can methylate the C5 position of the second cytosine in CCAGG and CCTGG sequences. ^d^ dam denotes sites modified by the site-specific DNA methyltransferase Dam, which can methylate the N6 position of adenine in GATC sequences. ^e^ Numbers represent the total sites detected by ONT sequencing, including both methylated and unmethylated sites. ^f^ Average methylation rate is calculated as the number of methylated reads divided by the sum of methylated and unmethylated reads at each site, averaged across all detected sites.

**Table 2:** Nine R-M systems in *C. huifangae* SJ-1 annotated in REBASE.

No.	Type	Function ^a^	REBASE Gene id	NCBI Locus_tag
1	I	MT	M.ChuSJ1ORFCP	FKL89_RS10995
		S	S.ChuSJ1ORFCP	FKL89_RS11000
		RE	ChuSJ1ORFCP	FKL89_RS11015
2	I	MT	M.ChuSJ1ORFDP	FKL89_RS15195
		S	S.ChuSJ1ORFDP	FKL89_RS15185
		RE	ChuSJ1ORFDP	FKL89_RS15175
3	II	MT	M1.ChuSJ1ORFEP	FKL89_RS15055
			M2.ChuSJ1ORFEP	FKL89_RS15060
		(Type IIG)MT/RE	ChuSJ1ORFEP	FKL89_RS15065
4	II	(Type IIG)MT/RE	ChuSJ1ORFBP	FKL89_RS01260
5	II	MT	M.ChuSJ1ORFHP	FKL89_RS01580
		RE	ChuSJ1ORFHP	FKL89_RS01585
6	II	MT	M.ChuSJ1ORFAP	FKL89_RS01745
		V	V.ChuSJ1ORFAP	FKL89_RS01740
7	II	MT	M.ChuSJ1ORFFP	FKL89_RS06620
8	II	MT	M.ChuSJ1ORFIP	FKL89_RS00880
9	III	MT	M.ChuSJ1ORFGP	FKL89_RS03395
		RE	ChuSJ1ORFGP	FKL89_RS03385

^a^ MT, methyltransferase; S, specificity subunit (for Type I R-M systems); RE, restriction endonuclease; (Type IIG)MT/RE, bifunctional fusion protein possessing both methyltransferase and restriction endonuclease activities; V, very short patch repair endonuclease.

**Table 3:** Nine specific targeting motifs of MTases from *C. huifangae* SJ-1.

No.	Motif ^a^	CenterPos	Type of Modification	Fraction	nDetected	nGenome
1	CC**A**GCC	3	6mA	1.0	6202	6202
2	RG**A**TCY	3	6mA	1.0	3866	3866
3	GGC**C**CG	4	4mC	0.999	4061	4063
4	TCAG**C**GC	5	4mC	0.999	2833	2835
5 ^b^	AGC**A**NNNNNNNCTC	4	6mA	1.0	829	829
6 ^b^	G**A**GNNNNNNNTGCT	2	6mA	1.0	829	829
7 ^b^	CA**A**YNNNNCTC	3	6mA	1.0	505	505
8 ^b^	G**A**GNNNNRTTG	2	6mA	1.0	505	505
9	AGTCG**A**CT	6	6mA	0.991	329	332

^a^ Motif: motif sequence; CenterPos: position of modification within the motif; Type of modification: type of base modification; Fraction: percentage of the base modification found in the genome; nDetected: number of modified motifs; nGenome: number of this motif in the genome. ^b^ For motifs 5 and 6, as well as motifs 7 and 8, the two sequences within each pair are reverse complements of each other and therefore represent the same targeting sequence read from opposite strands.

## Data Availability

The complete methylome sequences of *C. huifangae* SJ-1 have been deposited in the Genome Sequence Archive (GSA) at the China National Center for Bioinformation (CNCB) under the accession numbers CRX2955868 (ONT) and CRX2956052 (SMRT), respectively.

## Supplementary Materials

The following supporting information can be downloaded at https://www.mdpi.com/article/10.3390/microorganisms14081666/s1. Table S1: Strains and plasmids used in this study; Table S2: Broad-host-range plasmids used in this study; Table S3: The primers used in this study; Table S4: Additional anti-phage defense systems predicted by DefenseFinder in *C. huifangae* SJ-1 in addition to R‑M systems; Table S5: Distribution of methyltransferase-specific targeting motifs of *C. huifangae* SJ-1 on plasmid pBBR1MCS-2; Figure S1: Transformation of pBBR1MCS-2 into *C. huifangae* SJ-1; Figure S2: Distribution of methylation modification levels of each type in the genome of *C. huifangae* SJ-1; Figure S3: Characterization of the de‑immunized pBBR1MCS‑2 plasmid in *C. huifangae* SJ‑1; Figure S4: Effect of motif 6 sequence variations on RM‑C mediated cleavage; Sequence S1: Nucleotide sequence of the de‑immunized pBBR1MCS‑2 plasmid; Supplementary Sequencing Data: Sanger sequencing verification of reporter plasmids. References [38,39,40,41,42,43] are cited in the supplementary materials.
