# Supplementary material for: Genome-Wide DNA Methylation and Restriction-Modification Systems in Casimicrobium huifangae SJ-1, One of the Core Bacteria in Activated Sludge
Source: Microorganisms. 2026 Jul 30;14(8):1666. doi: 10.3390/microorganisms14081666 (PMC13515534; doi:10.3390/microorganisms14081666)

Supplementary materials for

Genome-Wide DNA Methylation and Restriction-Modification Systems in *Casimicrobium huifangae* SJ-1, One of the Core Bacteria in Activated Sludge

Jihong Yi [1,†], Kaiyue Zhu [1,†], Ze-Shen Liu [2], Meng Si [1], Hong Sun [1], Haiyan Huang [3], He Jiang [1], Shuang-Jiang Liu [1,2] and Shuning Wang [1,*]

[1] State Key Laboratory of Microbial Technology, Microbial Technology Institute, Shandong University, Qingdao 266237, China; jihongyi@sdu.edu.cn (J.Y.); 18252759628@163.com (K.Z.); 15100165972@163.com (M.S.); shsdauwine1@163.com (H.S.); jianghe@sdu.edu.cn (H.J.); liusj@sdu.edu.cn (S.L.)

[2] State Key Laboratory of Microbial Diversity and Innovative Utilization, Environmental Microbiology Research Center, Institute of Microbiology, Chinese Academy of Sciences, Beijing, 100101, China; liuzs@im.ac.cn (Z.L.)

[3] Department of Pathogen Biology, School of Clinical and Basic Medicine, Shandong First Medical University & Shandong Academy of Medical Sciences, Jinan 250117, China; yanhh2006@163.com

* Correspondence: shuningwang@sdu.edu.cn

[†] These authors contributed equally to this work

This file includes:
Table S1-S5
Figure S1-S4
Sequence 1
Supplementary Sequencing Data

Table S1. Strains and plasmids used in this study.

Strain or plasmid	Description	Source
Strain		
C. huifangae SJ-1	G^-, wild type	[15]
C. huifangae SJ-1-mcs2	*C. huifangae* SJ-1 strain harboring pBBR1MCS-2	This study
E. coli GB05-dir	F^- Δ(*fhu*A) Δ(*rec*ET) Δ(*ybc*C) attB::PBAD-ETγA (*rec*E^+ *rec*T^+ *red*γ^+ *rec*A^+) *ara*C^+ *hsd*R514 Δ(*ara*BAD) Δ(*lac*Z) *rps*L^+ *end*A^+	[16]
E. coli BL21(DE3)	F^- *omp*T *gal* dcm lon *hsd*SB(r_B^- m_B^-) λ(DE3 [*lac*I *lac*UV5-T7 *gene* 1 *ind*1 *sam*7 *nin*5])	Novagen
E. coli DH5α	F^- φ80d *lac*ZΔM15 Δ(*lac*ZYA-*arg*F) U169 *end*A1 *rec*A1 *hsd*R17(r_k^- m_k^+) *sup*E44λ^- *thi*-1 *gyr*A96 *rel*A1 *pho*A	Novagen
E. coli JM110	*rps*L (Str^R) *thr leu thi*-1 *lac*Y *gal*K *gal*T *ara ton*A *tsx dam dcm sup*E44Δ(*lac-pro*AB) /F[*tra*D36 *pro*AB *lac*I^q*lac*ZΔM15]	Novagen
E. coli BL21(DE3)-C-M/S	*E. coli* BL21(DE3) containing plasmid pET-28b (+)-C-*hsd*M/S	This study
E. coli JM110- pMotif6-1	*E. coli* JM110 containing plasmid pMotif6-1	This study
E. coli JM110- pMotif6-2	*E. coli* JM110 containing plasmid pMotif6-2	This study
E. coli JM110- pMotif6-3	*E. coli* JM110 containing plasmid pMotif6-3	This study
E. coli JM110- pMotif6-4	*E. coli* JM110 containing plasmid pMotif6-4	This study
E. coli JM110- pMotif8-1	*E. coli* JM110 containing plasmid pMotif8-1	This study
Plasmid		
pET-28b (+)	Kanamycin resistance, high copy number *E. coil* expressing vector	Novagen
pET-28b (+)-C-*hsd*M/S	pET-28b(+) derivative containing M.ChuSJ1ORFCP and S.ChuSJ1ORFCP	This study
pTrc99a	Ampicillin resistance, IPTG-inducible trc promoter expression vector	Novagen
pTrc99a-C-*hsd*R	pTrc99a derivative containing ChuSJ1ORFCP	This study
pMotif6-1	pET-28b(+) backbone with native motif6 and motif8 sites eliminated, then engineered to carry	This study

	a single motif6 sequence: 5′-GAGATCCGGCTGCT-3′.	
pMotif6-2	pMotif6-1 derivative with the motif6 sequence mutated to 5′-GAGGCGGGGCTGCT-3′	This study
pMotif6-3	pMotif6-1 derivative with the motif6 sequence mutated to 5′- GAGATATTCCTGCT-3′	This study
pMotif6-4	pMotif6-1 derivative with the motif6 sequence mutated to 5′- GAGATCCGGCAGCT-3′	This study
pMotif8-1	pET-28b(+) backbone with native motif6 and motif8 sites eliminated, then engineered to carry a single motif8 sequence: 5′-GAGGGGAATTG-3′.	This study
de-immunized pBBR1MCS-2	Derived from pBBR1MCS-2, whose methylation recognition sites have been mutated/modified	This study

Table S2. Broad-host-range plasmids used in this study.

Plasmid	Replicon	Selective marker (working concentration)	Reference
pBBR1MCS-2	pBBR1	Km^r (50 μg/mL)	[38]
pMMB66	RSF1010	Ap^r (50 μg/mL)	[39]
pRL1342	RSF1010	Cm^r (25 μg/mL)	[40]
pRKISC	RK2	Tc^r (25 μg/mL)	[41]
pJB653	RK2	Ap^r (50 μg/mL)	[42]
pKT247	R300B	Ap^r (50 μg/mL)	[43]

Table S3. The primers used in this study.

Primers	Sequence (5′ - 3′)
Primers for qRT-PCR	
f-AP-M	GGTGTAGAGCGGGACAACAA
r-AP-M	TGGTCCGCCAATCACTATCG
f-BP	CTTGGACATTGGGTTGTGCG
r-BP	GACATCGTGGAAATGCTGGC
f-CP-M	ATGTTCGTGCAGAGCGAGAA
r-CP-M	GCTTTCCTGCCCGTAAATGG
f-CP-R	CTACGACGAGGTCATCAGGC
r-CP-R	TAGATATGCGGTCGCAGCAG
f-DP-M	GTGCGGATCGGGAAGTTTTG
r-DP-M	AGAAGGACCGCTTCGACATC
f-DP-R	ATGGTGTTCACTGGTCGCAT
r-DP-R	GATCCTGACCACCTCGCAAA
f-EP-M1	CGATCACTTTTGCGGAGTGG
r-EP-M1	AACTTGGATGAGCCGGTGAG
f-EP-M2	CGCTTGAGCCACGCTATTTG
r-EP-M2	TCGAATGTGGTCGTCTGCAA
f-EP-R	CTGCCGTACTTGCAGCATTC
r-EP-R	CTTTTCGGCGAGCAGATTCG
f-FP-M	CCCTATGTGCGTCACCATCA
r-FP-M	CAGATGCAACCCTGCCATTG
f-GP-M	GCGTTCAAACCACGAAACGA
r-GP-M	GGTGGCCATCAACACACAAC
f-GP-R	GTTCGGCACCCAATTGCTTT
r-GP-R	TACTCGACGTTGAACAGCCC
f-HP-M	GACATAAAGGGGCTGCCTGT
r-HP-M	AAACCCCCAAGCCTGCATTA
f-HP-R	GCAATTTCTCCCAGTGCGTG

r-HP-R	AGAGCGAACAGGGCAAAAGA
f-IP	CTGCATCCGGCTTTTTCTCG
r-IP	ACACCCTGCAAGTACCCAAG
f-SJ-16S	CACCTGTGCCCAGATTCCTT
r-SJ-16S	AAGCGGTGGATGATGTGGTT
f-km	CAAGATGGATTGCACGCAGG
r-km	GCAGCCGATTGTCTGTTGTG
Primers for plasmid construction	
f-C-*hsd*M/S	TCTAGAAATAATTTTGTTTAACTTTAAGAAGGAGATATAATGCACCAC CACCACCACCACATGATCGAAGACATCCGCAA
r-C-*hsd*M/S	GTTAGCAGCCGGATCTCAGTGGTGGTGGTGGTGGTGGAATTCTGCCG CCAACGCCTCTGCCT
f-C-*hsd*M/S-m1	CAGCCATATGGCTAGCATGACTGGTGGACAGCAAATGGGTATGATCG AAGACATCCGCAA
r-C-*hsd*M/S-m1	GGCTTTGTTAGCAGCCGGATCTCAGTGGTGGTGGTGGTGGTGAGCAA ACCGAGCTCGTGCCGCCAACGCCTCTGCCTCGG
f-C-*hsd*M/S-m2	CAGCCATATGGCTAGCATGACTGGTGGACAGCAAATGGGTATGATCG AAGACATCCGCAA
r-C-*hsd*M/S-m2	GGGCTTTGTTAGCAGCCGGATCTCAGTGGTGGTGGTGGTGGTGCAAT AGCTCTCGAGGTGCCGCCAACGCCTCTGCCTCG
ptrc99a-F	GGTCTGTTTCCTGTGTGAAA
ptrc99a-R	ATCATCATCATCATCACTAGGGCTGTTTTGGCGGATGAGA
11015-F	TTTCACACAGGAAACAGACCATGACCGAAGACCAGCTCGA
11015-R	CTAGTGATGATGATGATGATGCGACCAGGCGTCGCCCAAC
28b-F	GCTGAGCAATAACTAGCATA
28b-R	CGTATTAATTTCGCGGGATC
motif6-1-F	TATGCTAGTTATTGCTCAGCGGTGGCAGCAGCCAACTCAGCTTCCTTT CGGGCTTTGTTGAGATCCGGCTGCT

motif6-1-R	GATCCCGCGAAATTAATACGACTCACTATAGGGGAATTGTGAGCGGATAAAGCAGCCGGATCTCAACAAA
motif6-2-F	TATGCTAGTTATTGCTCAGCGGTGGCAGCAGCCAACTCAGCTTCCTTTCGGGCTTTGTTGAGGCGGGGCTGCT
motif6-2-R	GATCCCGCGAAATTAATACGACTCACTATAGGGGAATTGTGAGCGGATAAAGCAGCCCCGCCTCAACAAA
motif6-3-F	TATGCTAGTTATTGCTCAGCGGTGGCAGCAGCCAACTCAGCTTCCTTTCGGGCTTTGTTGAGATATTCCTGCT
motif6-3-R	GATCCCGCGAAATTAATACGACTCACTATAGGGGAATTGTGAGCGGATAAAGCAGGAATATCTCAACAAA
motif6-4-F	TATGCTAGTTATTGCTCAGCGGTGGCAGCAGCCAACTCAGCTTCCTTTCGGGCTTTGTTGAGATCCGGCAGCT
motif6-4-R	GATCCCGCGAAATTAATACGACTCACTATAGGGGAATTGTGAGCGGATAAAGCTGCCGGATCTCAACAAA
motif8-1-F	TATGCTAGTTATTGCTCAGCGGTGGCAGCAGCCAACTCAGCTTCCTTTCGGGCTTTGTTGAGGGGAATTG
motif8-1-R	GATCCCGCGAAATTAATACGACTCACTATAGGGGAATTGTGAGCGGATAACAATTCCCCTCAACAAAGCC

Table S4. Additional anti-phage defense systems predicted by DefenseFinder in *C. huifangae* SJ-1 in addition to R-M systems.

sys_id	Type	subtype	sys_beg	sys_end
CP041352.1_Lamassu-Mrr_6	Lamassu-Fam	Lamassu-Mrr	CP041352.1_138	CP041352.1_140
CP041352.1_BREX_III_3	BREX	BREX_III	CP041352.1_173	CP041352.1_182
CP041352.1_PD-T7-2_9	PD-T7-2	PD-T7-2	CP041352.1_179	CP041352.1_180
CP041352.1_DS-8_4	DS-8	DS-8	CP041352.1_651	CP041352.1_651
CP041352.1_PD-T4-3_7	PD-T4-3	PD-T4-3	CP041352.1_1123	CP041352.1_1123
CP041352.1_AbiP2_1	AbiP2	AbiP2	CP041352.1_1378	CP041352.1_1378
CP041352.1_Gabija_5	Gabija	Gabija	CP041352.1_2078	CP041352.1_2079
CP041352.1_PD-T4-6_8	PD-T4-6	PD-T4-6	CP041352.1_2172	CP041352.1_2172

Table S5. Distribution of methyltransferase-specific targeting motifs of *C. huifangae* SJ-1 on plasmid pBBR1MCS-2.

Motif	Number
CCAGCC	15
RGATCY	7
GGCCCG	6
TCAGCGC	1
AGCANNNNNNNCTC	1
GAGNNNNNNNTGCT	1
GAGNNNNRTTG	0
CAAYNNNNCTC	0
AGTCGACT	0

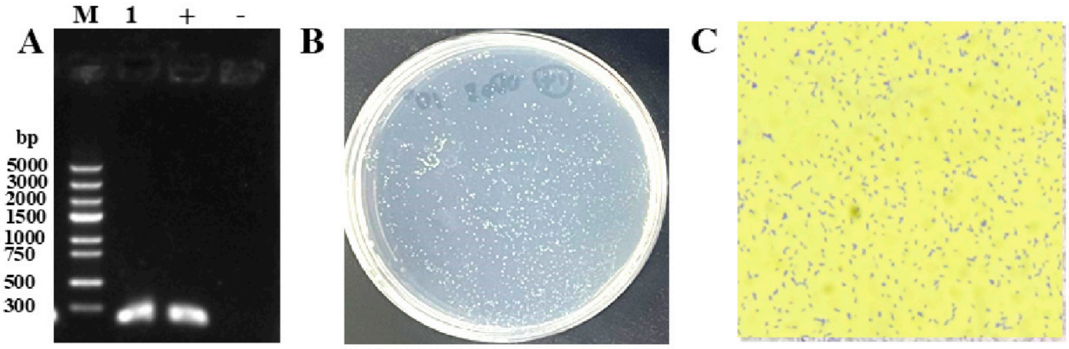

Figure S1. Transformation of pBBR1MCS-2 into *C. huifangae* SJ-1. (A) PCR verification of transformant SJ-1-mcs2 using the universal primers M13F/M13R. Lane M: DNA marker; lane 1: transformant SJ-1-mcs2; lane +: positive control (pBBR1MCS-2 plasmid); lane −: negative control (no template or wild type strain SJ-1). (B) plate culture of transformant SJ-1-mcs2. (C) cell morphology (10×/100× [oil]) under the microscope.

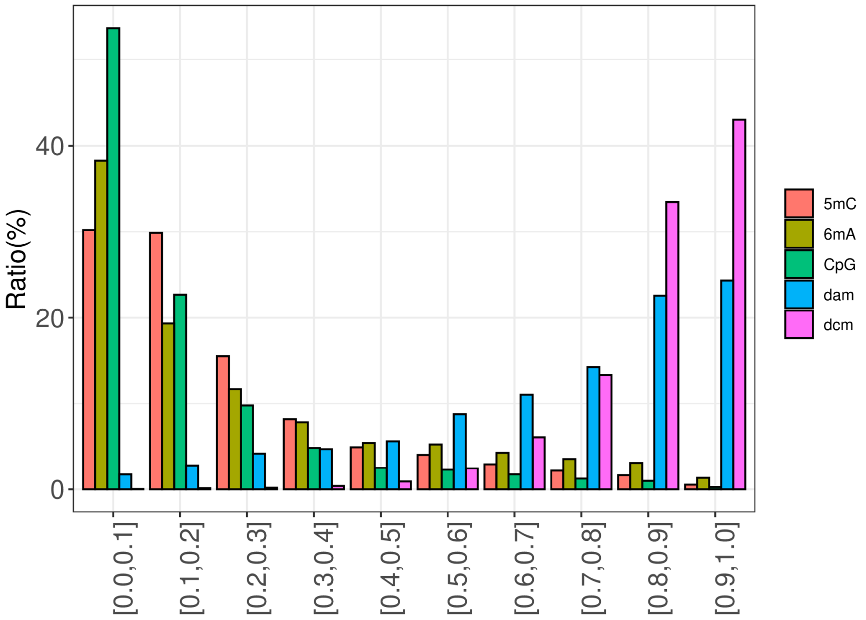

Figure S2. Distribution of methylation modification levels of each type in the genome of *C. huifangae* SJ-1.

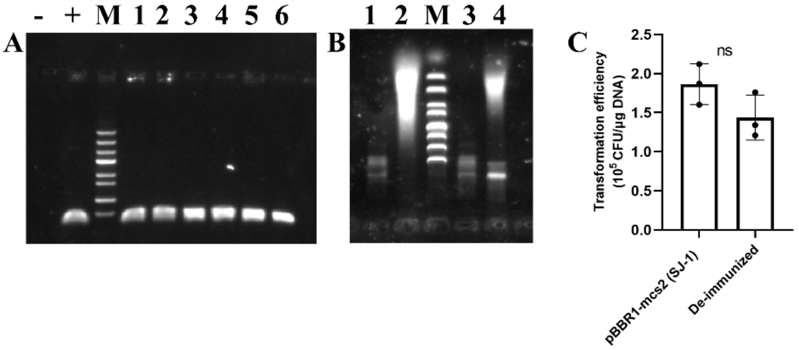

Figure S3. Characterization of the de-immunized pBBR1MCS-2 plasmid in *C. huifangae* SJ-1. **(A)** PCR verification of transformants harboring the de-immunized pBBR1MCS-2 plasmid. Lane M, DNA marker; lane (−), negative control (no template); lane (+), positive control (purified mutant plasmid DNA); lanes 1–6, six independent transformants. The expected PCR product was detected in all six transformants using the universal primers M13F/M13R, confirming successful introduction of the de-immunized plasmid into *C. huifangae* SJ-1. These transformants were stably maintained under kanamycin selection and consistently yielded the PCR product over successive passages. **(B)** Agarose gel electrophoresis detecting the degradation of pBBR1MCS-2 and de-immunized pBBR1MCS-2 plasmids upon incubation with crude cell lysates of *C. huifangae* SJ-1. Lane M, DNA marker; lane 1, pBBR1MCS-2 (from *E. coli*) without lysate; lane 2, pBBR1MCS-2 (from *E. coli*) with lysate; lane 3, de-immunized pBBR1MCS-2 (from *E. coli*) without lysate; lane 4, de-immunized pBBR1MCS-2 (from *E. coli*) with lysate. The unmodified pBBR1MCS-2 was completely degraded upon lysate treatment (lane 2), whereas the de-immunized plasmid remained intact (lane 4). **(C)** Comparison of transformation efficiency (CFU/μg DNA) between pBBR1MCS-2 extracted from the transformant SJ-1-mcs2 and de-immunized pBBR1MCS-2 extracted from *E. coli*. Data are shown as mean ± SD (n = 3 biologically independent transformations). ns, not significant (Student's t-test).

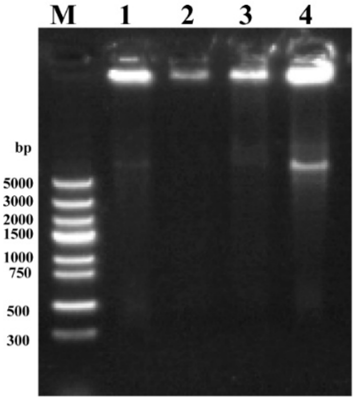

Figure S4. Effect of motif 6 sequence variations on RM-C mediated cleavage. Reporter plasmids carrying different motif 6 variants were incubated with the pre-assembled MSR complex. Lane 1, pMotif6-1 (N_7=ATCCGGC); lane 2, pMotif6-2 (N_7 =

GCGGGGC); lane 3, pMotif6-3 (N_7 = ATATTCC); lane 4, pMotif6-4 (3′-terminal TGCT replaced with AGCT). All three pMotif6-1/-2/-3 variants were completely degraded by MSR complex, whereas the pMotif6-4 plasmid remained intact. These results indicate that the RM-C system tolerates sequence variation in the seven-base spacer but strictly requires the conserved 5′-GAGN_7TGCT-3′. M, DNA marker.

Sequence S1. Nucleotide sequence of the de-immunized pBBR1MCS-2 plasmid. Mutated bases are highlighted in bold.

ctcggTgccgtctcttgggcttgatcggccttcttgcgcatctcacgcgctcctgcggcggcctgtagggcaggctcatac
ccctgccgaaccgcttttgtcagccggtcggccacggcttccggcgtctcaacgcgctttgagattcccagcttttcggcca
atccctgcggtgcataggcgcgtggctcgaccgcttgcgggctgatggtgacgtggcccactggtggccgctccagggc
ctcgtagaacgcctgaatgcgcgtgtgacgtgccttgctgccctcgatgccccgttgcagccctagatcggccacagcgg
ccgcaaacgtggtctggtcgcgggtcatctgcgctttgttgccgatgaactccttggccgacagcctgccgtcctgcgtcag
cggcaccacgaacgcggtcatgtgcgggc**G**tggtttcgtcacggtggatgctggccgtcacgatgcgatccgccccgta
cttgtccgccag**G**ccacttgtgcgccttctcgaagaacgccgcctgctgttcttggc**G**tggccgacttccaccattccggg
c**G**tggccgtcatgacgtactcgaccgccaacacagcgtccttgcgccgcttctctggcagcaactcgcgcagtcggccc
atcgcttcatcggtgctgctggccgcccagtgctcgttctctggcgtcctgctggcgtcagcgttgggcgtctcgcgctcgc
ggtaggcgtgcttgagactggccgccacgttgcccattttcgccagcttcttgcatcgcatgatcgcgtatgccgccatgcct
gcccctcccttttggtgtccaaccggctcgacgggggcagcgcaaggcggtgcctccggcgg**T**gccactcaatgcttga
gtatactcactagactttgcttcgcaaagtcgtgaccgcctacggcggctgcggcgccctacgggcttgctctccgggcttc
gccctgcgcggtcgctgcgctcccttgccaggc**A**cgtggatatgtggacgatggccgcgagcggccaccggct**T**gctc
gcttcgctcggc**T**cgtggacaaccctgctggacaagctgatggacaggctgcgcctgcccacgagcttgaccacaggg
attgcccaccggctacccagc**T**ttcgaccacatacccaccggctccaactgcgcggcctgcggccttgccccatcaatttt
tttaattttctctggggaaaagcctccggcctgcggcctgcgcgcttcgcttgccggttggacaccaagtggaaggcgggtc
aaggctcgcgcagcgaccgcgcagcggcttggccttgacgcgcctggaacgacccaagcctatgcgagtgggggcagt
cgaaggcgaagcccgcccgcctgccccccgagcctcacggcggcgagtgcgggggttccaagggggcagcgccacc
ttgggcaaggccgaaggccgcgcagtcgatcaacaagccccggaggggccactttttgccggagggggagccgcgcc
gaaggcgtgggggaaccccgcaggggtgcccttctttgggcaccaaagaactagatatagggcgaaatgcgaaagactt
aaaaatcaacaacttaaaaaagggggggtacgcaacagctcattgcggcaccccccgcaatagctcattgcgtaggttaaa
gaaaatctgtaattgactgccacttttacgcaacgcataattgttgtcgcgctgccgaaaagttgcagctgattgcgcatggtg
ccgcaaccgtgcggcaccctaccgcatggagataagcatggccacgcagtccagagaaatcggcattcaagccaagaa
caagcccggtcactgggtgcaaacggaacgcaaagcgcatgaggcgtgggccgggcttattgcgaggaaacccacgg
cggcaatgctgctgcatcacctcgtggcgcagatgggccaccagaacgccgtggtggtcagccagaagacactttccaa
gctcatcggacgttctttgcggacggtccaatacgcagtcaaggacttggtggccgagcgctggat**A**tccgtcgtgaagct
caacggccccggcaccgtgtcggcctacgtggtcaatgaccgcgtggcgtggggcca**A**ccccgcgaccagttgcgcct
gtcggtgttcagtgccgccgtggtggttgatcacgacgaccaggacgaatcgctgttggggcatggcgacctgcgccgca
tcccgaccctgtatccgggcgagcagcaactaccgaccggccccggcgaggagccgcccag**T**cagcccggcattccg
ggcatggaaccagacctgcc**G**gccttgaccgaaacggaggaatgggaacggcgcgggcagcagcgcctgccgatgc
ccgatgagccgtgttttctggacgatggcgagccgttggagccgccgacacgggtcacgctgccgcgccggtagcactt
gggttgcgcagcaacccgtaagtgcgctgttccagactatcggctgtagccgcctcgccgccctataccttgtctgcctccc
cgcgttgcgtcgcggtgcatggagccg**A**gccacctcgacctgaatggaagccggcggcacctcgctaacggattcacc
gtttttatcaggctctgggaggcagaataaatgatcatatcgtcaattattacctccacggggagagcctgag**A**caaactgg
cctcaggcatttgagaagcacacggtcacactgcttccggtagtcaataaaccggtaaaccagcaatagacataagcggct
atttaacgaccctgccctgaaccgacgaccgggtcgaatttgctttcgaatttctgccattcatccgcttattatcacttattcag
gcgtagcaccaggcgtttaagggcaccaataactgccttaaaaaaattacgccccgccctgccactcatcgcagtcggcct
attggttaaaaaatgagctgatttaacaaaaatttaacgcgaattttaacaaaatattaacgcttacaatttccattcgccattcag
gctgcgcaactgttgggaagggcgatcggtgcg**C**gcctcttcgctattacgccagctggcgaaagggggatgtgctgca
aggcgattaagttgggtaacgccagggttttcccagtcacgacgttgtaaaacgacggccagtgagcgcgcgtaatacga
ctcactatagggcgaattggagctccaccgcggtggcggccgctctagaactagtgg**G**tcccccgggctgcaggaattc
gatatcaagcttatcgataccgtcgacctcgaggggggg**C**cccggtacccagcttttgttccctttagtgagggttaattgcgc

gcttggcgtaatcatggtcatagctgtttcctgtgtgaaattgttatccgctcacaattccacacaacatacgagccggaagca
taaagtgtaaagcctggggtgcctaatgagtgagctaactcacattaattgcgttgcgctcactgcccgctttccagtcggga
aacctgtcgtgccagctgcattaatgaatcggccaacgcgcggggagaggcggtttgcgtattgggcgcatgcataaaaa
ctgttgtaattcattaagcattctgccgacatggaagccatcacaaacggcatgatgaacctgaatcgccagcggcatcagc
accttgtcgccttgcgtataatatttgcccatgggggtgggcgaagaactccagcatgaga**C**tccccgcgctggaggatca
tcca**G**gccggcgtcccggaaaacgattccgaagcccaacctttcatagaaggcggcggtggaatcgaaatctcgtgatg
gcaggttgggcgtcgcttggtcggtcatttcgaaccccagagtcccgctcagaagaactcgtcaagaaggcgatagaagg
cgatgcgctgcgaatcgggagcggcgataccgtaaagcacgaggaagcggtcagcccattcgccgccaagctcttcag
caatatcacgggtagccaacgctatgtcctgatagcggtccgccacacc**T**agccggccacagtcgatgaatccagaaaa
gcggccattttccaccatgatattcggcaagcaggcatcgccatgggtcacgacgag**G**tcctcgccgtcgggcatgcgc
gccttgagcctggcgaacagttc**A**gctggcgcgagcccctgatg**T**tcttcgtccagatcatcctgatcgacaagaccggc
ttccatccgagtacgtgctcgctcgatgcgatgtttcgcttggtggtcgaatgggcaggtagccggatcaagcgtatgcagc
cgccgcattgcatcagccatgatggatactttctcggcaggagcaaggtgagatgacaggag**G**tcctgccccggcacttc
gcccaatagcagccagtcccttcccgcttcagtgacaacgtcgagcacagctgcgcaaggaacgcccgtcgtggc**T**ag
ccacgatagccgcgctgcctcgtcctgcagttcattcagggcaccggacaggtcggtcttgacaaaaagaaccgggcgc
ccctg**G**gctgacagccggaacacggcggcatcagagcagccgattgtctgttgtgcccagtcatagccgaatagcctctc
cacccaagcggccggagaacctgcgtgcaatccatcttgttcaatcatgcgaaacgatcctcatcctgtctcttgatcag**G**t
cttgatcccctgcgccatcag**G**tccttggcggcaagaaagccatccagtttactttgcagggcttcccaaccttaccagagg
gcgccccagctggcaattccggttcgcttgctgtccataaaccgcccagtctagctatcgccatgtaagcccactgcaagc
tacctgctttctctttgcgcttgcgttttcccttgtccagatagcccagtagctgacattcatcccaggtggcacttttcggggaa
atgtgcgcgcccgcgttcctgctggcgctgggcctgtttctggcgctggacttcccgctgttccgtcagcagcttttcgccca
cggccttgatgatcgcggcggccttggcctgcatatcccgattcaacggccccagggcgtccagaacgggcttcaggcg
ctcccgaaggt

Supplementary Sequencing Data: Sanger sequencing verification of reporter plasmids. The motif sequences carried by each reporter plasmid are highlighted in gray.

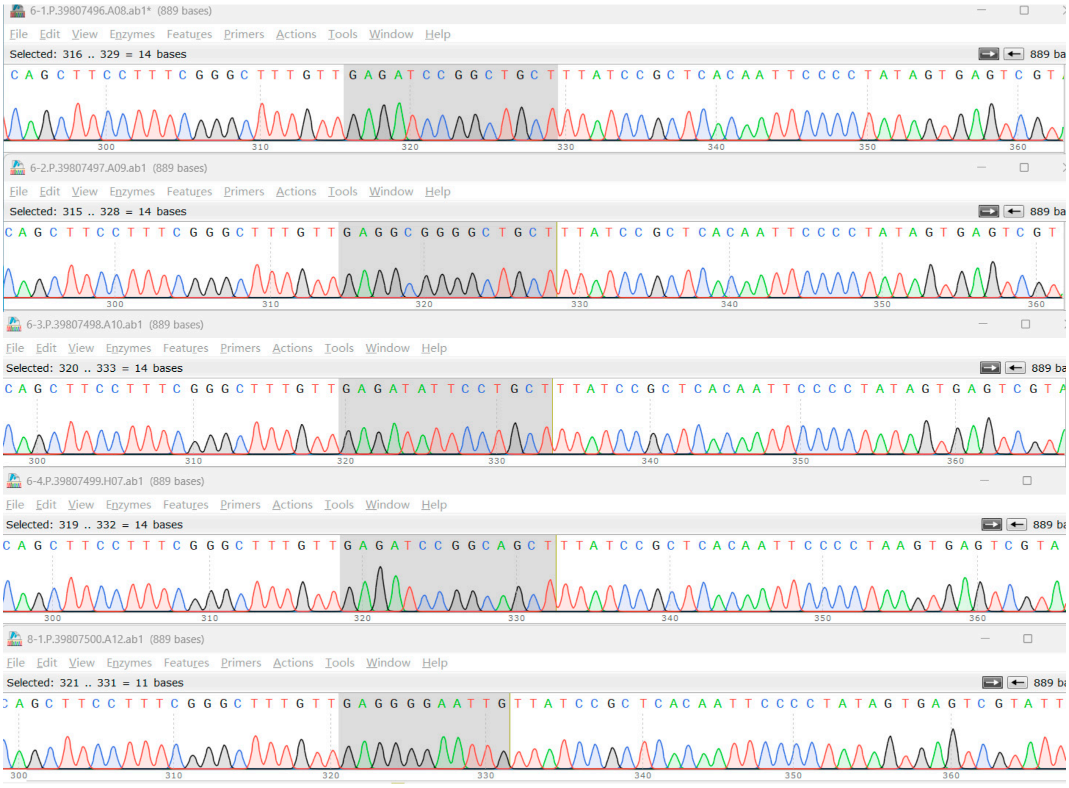

Supplement: Supplementary file 1 [file microorganisms-14-01666-s001.zip › microorganisms-4405156-supplementary.pdf]
